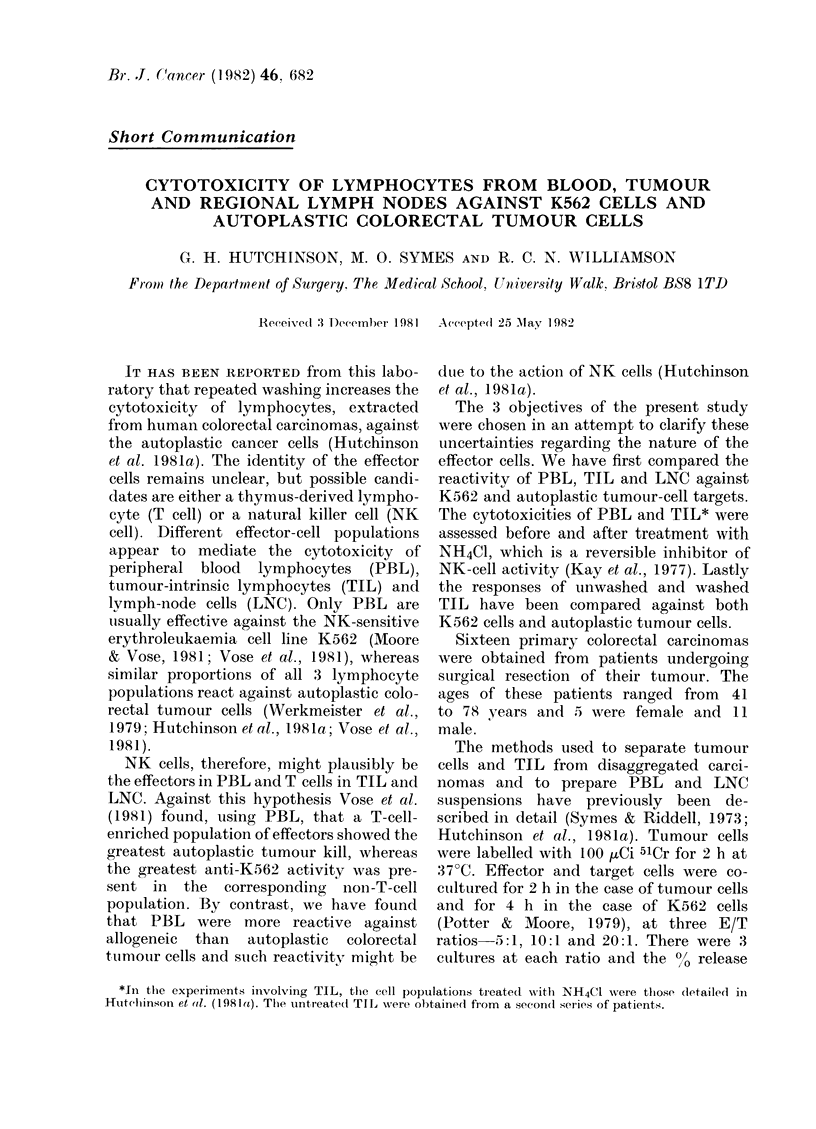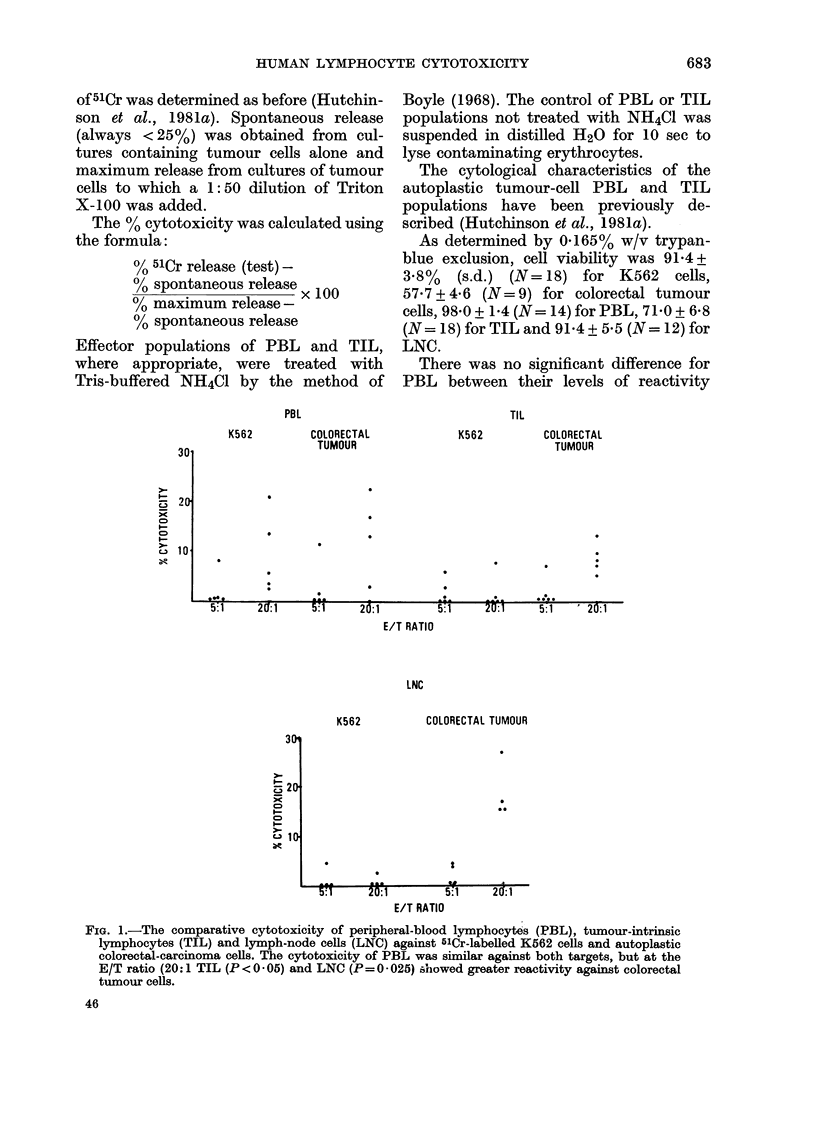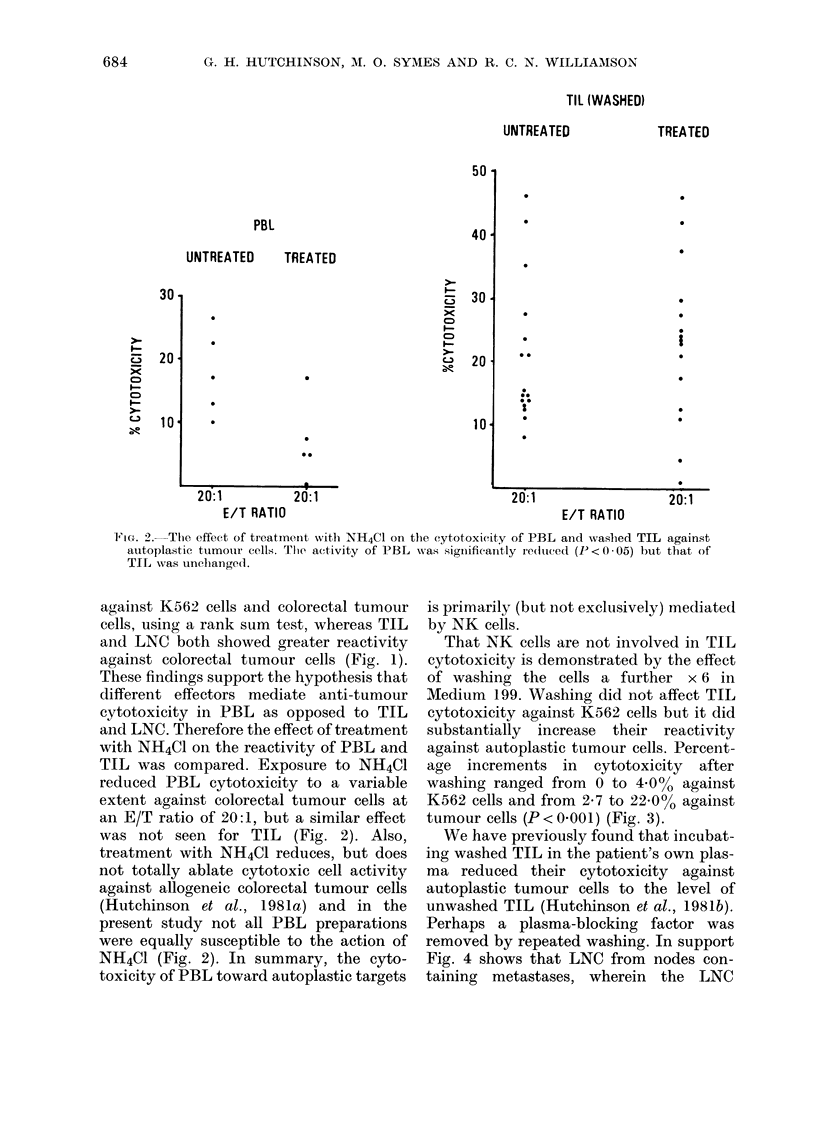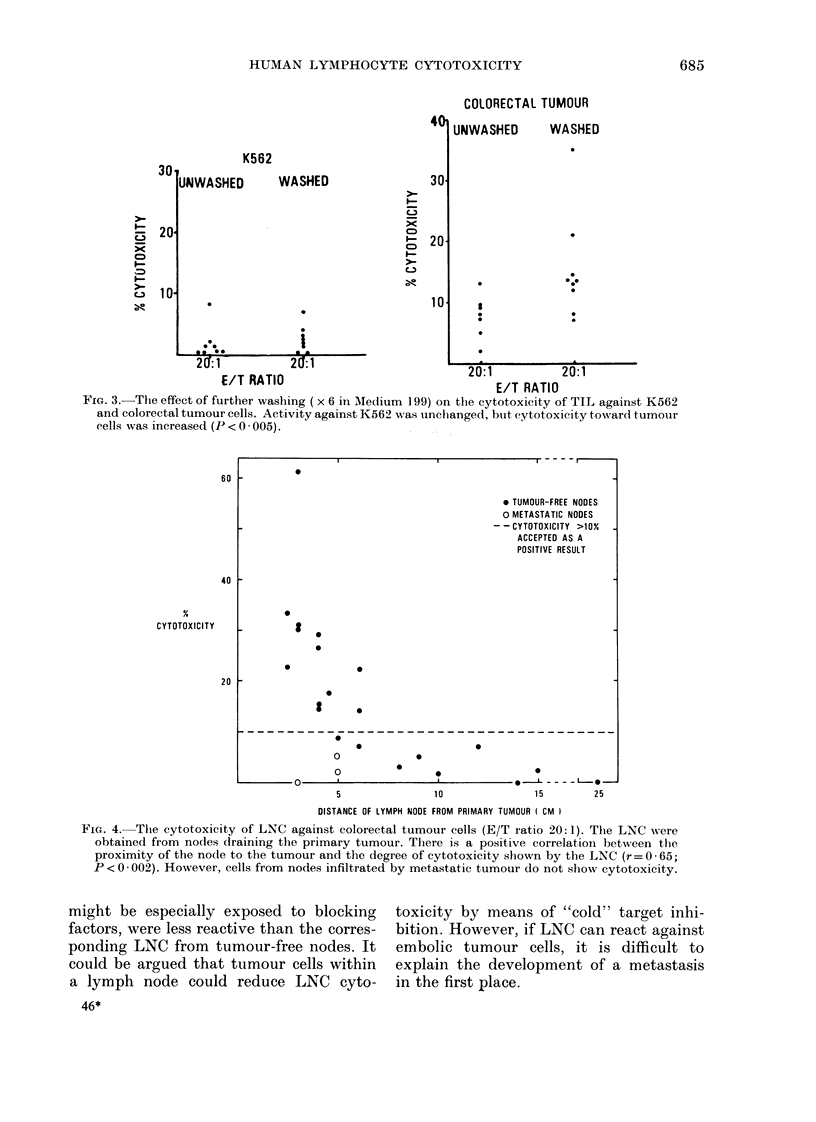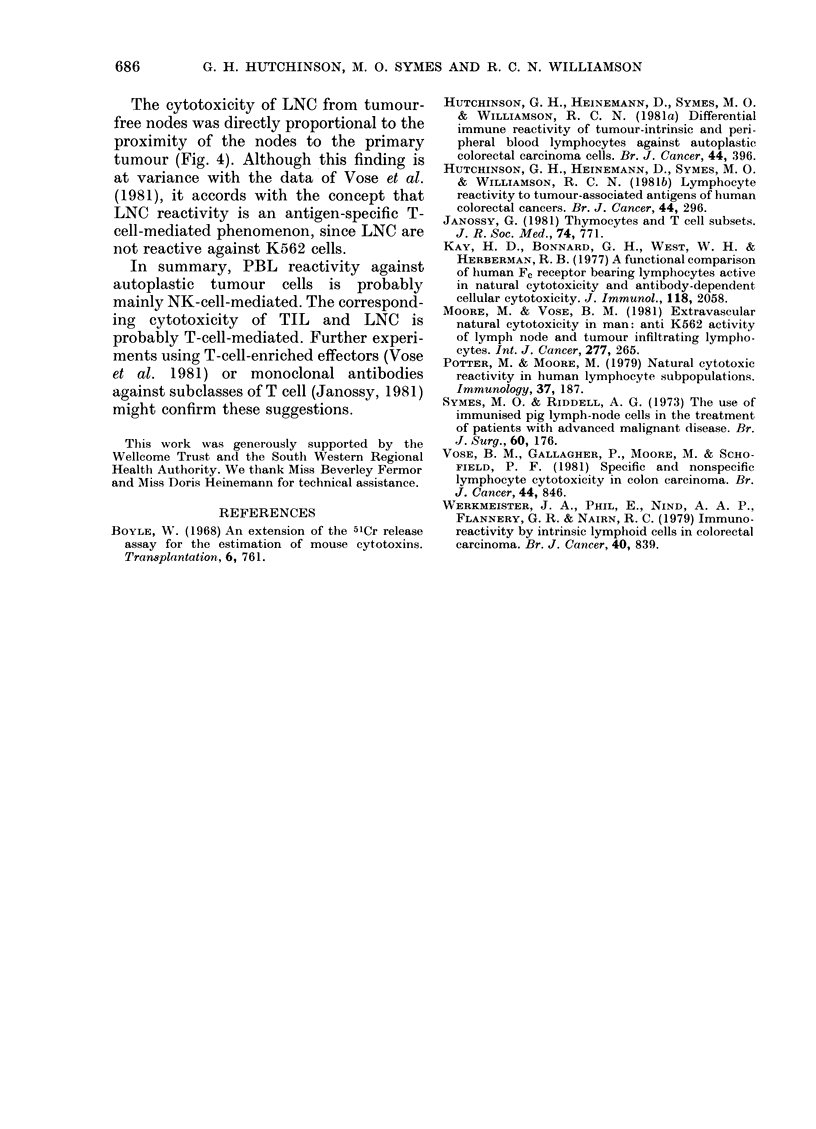# Cytotoxicity of lymphocytes from blood, tumour and regional lymph nodes against K562 cells and autoplastic colorectal tumour cells.

**DOI:** 10.1038/bjc.1982.255

**Published:** 1982-10

**Authors:** G. H. Hutchinson, M. O. Symes, R. C. Williamson


Br. J. Cancer (1982) 46, 682

### Short Communication

### CYTOTOXICITY OF LYMPHOCYTES FROM BLOOD, TUMOUR AND REGIONAL LYMPH NODES AGAINST K562 CELLS AND AUTOPLASTIC COLORECTAL TUMOUR CELLS

G. H. HUTCHINSON, M. O. SYMES AND R. C. N. WILLIAMSON

*From the Department of Surgery, The Medical School, University Walk, Bristol BS8 1TD*

Received 3 December 1981 Accepted 25 May 1982

IT HAS BEEN REPORTED from this laboratory that repeated washing increases the cytotoxicity of lymphocytes, extracted from human colorectal carcinomas, against the autoplastic cancer cells (Hutchinson *et al.* 1981*a*). The identity of the effector cells remains unclear, but possible candidates are either a thymus-derived lymphocyte (T cell) or a natural killer cell (NK cell). Different effector-cell populations appear to mediate the cytotoxicity of peripheral blood lymphocytes (PBL), tumour-intrinsic lymphocytes (TIL) and lymph-node cells (LNC). Only PBL are usually effective against the NK-sensitive erythroleukaemia cell line K562 (Moore & Vose, 1981; Vose *et al.*, 1981), whereas similar proportions of all 3 lymphocyte populations react against autoplastic colorectal tumour cells (Werkmeister *et al.*, 1979; Hutchinson *et al.*, 1981*a*; Vose *et al.*, 1981).

NK cells, therefore, might plausibly be the effectors in PBL and T cells in TIL and LNC. Against this hypothesis Vose *et al.* (1981) found, using PBL, that a T-cell-enriched population of effectors showed the greatest autoplastic tumour kill, whereas the greatest anti-K562 activity was present in the corresponding non-T-cell population. By contrast, we have found that PBL were more reactive against allogeneic than autoplastic colorectal tumour cells and such reactivity might be due to the action of NK cells (Hutchinson *et al.*, 1981*a*).

The 3 objectives of the present study were chosen in an attempt to clarify these uncertainties regarding the nature of the effector cells. We have first compared the reactivity of PBL, TIL and LNC against K562 and autoplastic tumour-cell targets. The cytotoxicities of PBL and TIL* were assessed before and after treatment with $NH_4Cl$, which is a reversible inhibitor of NK-cell activity (Kay *et al.*, 1977). Lastly the responses of unwashed and washed TIL have been compared against both K562 cells and autoplastic tumour cells.

Sixteen primary colorectal carcinomas were obtained from patients undergoing surgical resection of their tumour. The ages of these patients ranged from 41 to 78 years and 5 were female and 11 male.

The methods used to separate tumour cells and TIL from disaggregated carcinomas and to prepare PBL and LNC suspensions have previously been described in detail (Symes & Riddell, 1973; Hutchinson *et al.*, 1981*a*). Tumour cells were labelled with 100 $\mu$Ci $^{51}Cr$ for 2 h at 37°C. Effector and target cells were co-cultured for 2 h in the case of tumour cells and for 4 h in the case of K562 cells (Potter & Moore, 1979), at three E/T ratios—5:1, 10:1 and 20:1. There were 3 cultures at each ratio and the % release

*In the experiments involving TIL, the cell populations treated with $NH_4Cl$ were those detailed in Hutchinson *et al.* (1981*a*). The untreated TIL were obtained from a second series of patients.

of $^{51}Cr$ was determined as before (Hutchinson *et al.*, 1981*a*). Spontaneous release (always $<25\%$) was obtained from cultures containing tumour cells alone and maximum release from cultures of tumour cells to which a 1:50 dilution of Triton X-100 was added.

The % cytotoxicity was calculated using the formula:

$$\frac{\%\ ^{51}Cr\ \text{release (test)} - \%\ \text{spontaneous release}}{\%\ \text{maximum release} - \%\ \text{spontaneous release}} \times 100$$

Effector populations of PBL and TIL, where appropriate, were treated with Tris-buffered $NH_4Cl$ by the method of Boyle (1968). The control of PBL or TIL populations not treated with $NH_4Cl$ was suspended in distilled $H_2O$ for 10 sec to lyse contaminating erythrocytes.

The cytological characteristics of the autoplastic tumour-cell PBL and TIL populations have been previously described (Hutchinson *et al.*, 1981*a*).

As determined by 0·165% w/v trypan-blue exclusion, cell viability was $91{\cdot}4 \pm 3{\cdot}8\%$ (s.d.) ($N=18$) for K562 cells, $57{\cdot}7 \pm 4{\cdot}6$ ($N=9$) for colorectal tumour cells, $98{\cdot}0 \pm 1{\cdot}4$ ($N=14$) for PBL, $71{\cdot}0 \pm 6{\cdot}8$ ($N=18$) for TIL and $91{\cdot}4 \pm 5{\cdot}5$ ($N=12$) for LNC.

There was no significant difference for PBL between their levels of reactivity

FIG. 1.—The comparative cytotoxicity of peripheral-blood lymphocytes (PBL), tumour-intrinsic lymphocytes (TIL) and lymph-node cells (LNC) against $^{51}Cr$-labelled K562 cells and autoplastic colorectal-carcinoma cells. The cytotoxicity of PBL was similar against both targets, but at the E/T ratio (20:1 TIL ($P<0{\cdot}05$) and LNC ($P=0{\cdot}025$) showed greater reactivity against colorectal tumour cells.

FIG. 2.—The effect of treatment with $NH_4Cl$ on the cytotoxicity of PBL and washed TIL against autoplastic tumour cells. The activity of PBL was significantly reduced ($P < 0 \cdot 05$) but that of TIL was unchanged.

against K562 cells and colorectal tumour cells, using a rank sum test, whereas TIL and LNC both showed greater reactivity against colorectal tumour cells (Fig. 1). These findings support the hypothesis that different effectors mediate anti-tumour cytotoxicity in PBL as opposed to TIL and LNC. Therefore the effect of treatment with $NH_4Cl$ on the reactivity of PBL and TIL was compared. Exposure to $NH_4Cl$ reduced PBL cytotoxicity to a variable extent against colorectal tumour cells at an E/T ratio of 20:1, but a similar effect was not seen for TIL (Fig. 2). Also, treatment with $NH_4Cl$ reduces, but does not totally ablate cytotoxic cell activity against allogeneic colorectal tumour cells (Hutchinson *et al.*, 1981*a*) and in the present study not all PBL preparations were equally susceptible to the action of $NH_4Cl$ (Fig. 2). In summary, the cytotoxicity of PBL toward autoplastic targets is primarily (but not exclusively) mediated by NK cells.

That NK cells are not involved in TIL cytotoxicity is demonstrated by the effect of washing the cells a further $\times 6$ in Medium 199. Washing did not affect TIL cytotoxicity against K562 cells but it did substantially increase their reactivity against autoplastic tumour cells. Percentage increments in cytotoxicity after washing ranged from 0 to 4·0% against K562 cells and from 2·7 to 22·0% against tumour cells ($P < 0 \cdot 001$) (Fig. 3).

We have previously found that incubating washed TIL in the patient's own plasma reduced their cytotoxicity against autoplastic tumour cells to the level of unwashed TIL (Hutchinson *et al.*, 1981*b*). Perhaps a plasma-blocking factor was removed by repeated washing. In support Fig. 4 shows that LNC from nodes containing metastases, wherein the LNC

Fig. 3.—The effect of further washing ( × 6 in Medium 199) on the cytotoxicity of TIL against K562 and colorectal tumour cells. Activity against K562 was unchanged, but cytotoxicity toward tumour cells was increased ($P < 0 \cdot 005$).

Fig. 4.—The cytotoxicity of LNC against colorectal tumour cells (E/T ratio 20:1). The LNC were obtained from nodes draining the primary tumour. There is a positive correlation between the proximity of the node to the tumour and the degree of cytotoxicity shown by the LNC ($r = 0 \cdot 65$; $P < 0 \cdot 002$). However, cells from nodes infiltrated by metastatic tumour do not show cytotoxicity.

might be especially exposed to blocking factors, were less reactive than the corresponding LNC from tumour-free nodes. It could be argued that tumour cells within a lymph node could reduce LNC cytotoxicity by means of "cold" target inhibition. However, if LNC can react against embolic tumour cells, it is difficult to explain the development of a metastasis in the first place.

The cytotoxicity of LNC from tumour-free nodes was directly proportional to the proximity of the nodes to the primary tumour (Fig. 4). Although this finding is at variance with the data of Vose *et al.* (1981), it accords with the concept that LNC reactivity is an antigen-specific T-cell-mediated phenomenon, since LNC are not reactive against K562 cells.

In summary, PBL reactivity against autoplastic tumour cells is probably mainly NK-cell-mediated. The corresponding cytotoxicity of TIL and LNC is probably T-cell-mediated. Further experiments using T-cell-enriched effectors (Vose *et al.* 1981) or monoclonal antibodies against subclasses of T cell (Janossy, 1981) might confirm these suggestions.

This work was generously supported by the Wellcome Trust and the South Western Regional Health Authority. We thank Miss Beverley Fermor and Miss Doris Heinemann for technical assistance.

### REFERENCES

BOYLE, W. (1968) An extension of the $^{51}$Cr release assay for the estimation of mouse cytotoxins. *Transplantation*, **6**, 761.

HUTCHINSON, G. H., HEINEMANN, D., SYMES, M. O. & WILLIAMSON, R. C. N. (1981*a*) Differential immune reactivity of tumour-intrinsic and peripheral blood lymphocytes against autoplastic colorectal carcinoma cells. *Br. J. Cancer*, **44**, 396.

HUTCHINSON, G. H., HEINEMANN, D., SYMES, M. O. & WILLIAMSON, R. C. N. (1981*b*) Lymphocyte reactivity to tumour-associated antigens of human colorectal cancers. *Br. J. Cancer*, **44**, 296.

JANOSSY, G. (1981) Thymocytes and T cell subsets. *J. R. Soc. Med.*, **74**, 771.

KAY, H. D., BONNARD, G. H., WEST, W. H. & HERBERMAN, R. B. (1977) A functional comparison of human $F_c$ receptor bearing lymphocytes active in natural cytotoxicity and antibody-dependent cellular cytotoxicity. *J. Immunol.*, **118**, 2058.

MOORE, M. & VOSE, B. M. (1981) Extravascular natural cytotoxicity in man: anti K562 activity of lymph node and tumour infiltrating lymphocytes. *Int. J. Cancer*, **277**, 265.

POTTER, M. & MOORE, M. (1979) Natural cytotoxic reactivity in human lymphocyte subpopulations. *Immunology*, **37**, 187.

SYMES, M. O. & RIDDELL, A. G. (1973) The use of immunised pig lymph-node cells in the treatment of patients with advanced malignant disease. *Br. J. Surg.*, **60**, 176.

VOSE, B. M., GALLAGHER, P., MOORE, M. & SCHOFIELD, P. F. (1981) Specific and nonspecific lymphocyte cytotoxicity in colon carcinoma. *Br. J. Cancer*, **44**, 846.

WERKMEISTER, J. A., PHIL, E., NIND, A. A. P., FLANNERY, G. R. & NAIRN, R. C. (1979) Immunoreactivity by intrinsic lymphoid cells in colorectal carcinoma. *Br. J. Cancer*, **40**, 839.